# 

**Published:** 1851-05

**Authors:** 


### NOTICE TO SUBSCRIBERS.

With this number of the Journal we send out our bills including advance payment for the ensuing year Of course we wish to have them paid—by remittence. The Intelligencer will be sent regularly, every alternate month, to those only who have paid for the Journal including the current year's subscription.

---

### NOTICE TO READERS AND CORRESPONDENTS.

We have received several Original Papers, numerous Pamphle[illegible] Lectures, College Circulars, Reports, &c., which our want of ro[illegible] prevents an acknowledgement of in this number. We will try [illegible] reserve plenty of room hereafter, to acknowledge our obligations [illegible] such matters. In the mean time we return our hearty thanks [illegible] those who have remembered us in the distribution of their favors.

### RECEIPTS FOR THE JOURNAL FOR MARCH & APRIL.

| | |
|---|---|
| **ILLINOIS.** | |
| Drs. J. Bennett, | $2 00 |
| J. W. Freer, | 2 00 |
| W. W. Welch, | 3 00 |
| Harmon Wasson, | 2 00 |
| N. Plummer, | 3 00 |
| G. W. Minard, | 2 00 |
| R. R. Stone, | 2 00 |
| E. L. Patton, | 2 00 |
| E. L. Blanchard. | 2 00 |
| C. A. Hathwell, | 2 00 |
| F. Stevens, | 2 00 |
| H. Douglass, | 2 00 |
| D. Hess, | 4 00 |
| J. S. Bullock, | 2 00 |
| B. B. Everett, | 2 00 |
| O. Abbey, | 4 00 |
| Geo. Higgins, | 2 00 |
| Ira E. Oatman, | 2 00 |
| F. Ehrhardt, | 2 00 |
| W. H. Davis, | 4 00 |
| Giles P. Ransom, | 2 00 |
| A. E. Ames, | 2 00 |
| N. P. Holden, | 2 00 |
| J. S. Whitmire, | 2 00 |
| Samuel Wiley, | 2 00 |
| H. Hooker, | 8 00 |
| E. Ingalls, | 2 00 |
| C. C. Bradley, | 4 00 |
| **WISCONSIN.** | |
| Drs. Geo. D. Wilber, | 2 00 |
| C. C. Warner, | 2 00 |
| W. W. Blackman, | 2 00 |
| B. F. Mills, | 5 00 |
| J. M. Evans, | 2 00 |
| J. C. Mills, | 3 0 |
| Drs. Ira R. Rood, | $2 00 |
| W. Wadsworth, | 2 00 |
| N. L. Eastman, | 3 00 |
| R. Dunlap. Jr., | 2 00 |
| Jno. Philips, | 2 00 |
| Stodard G. Harvey, | 1 00 |
| **INDIANA.** | |
| Drs. J. H. Wiley, | 2 00 |
| P. Q. Stryker, | 2 00 |
| J. T. Mayfield, | 3 00 |
| W. Brenton, | 2 00 |
| T. L. Catherwood, | 3 25 |
| W. Townsend, | 2 00 |
| W. Matthews, | 2 00 |
| M. C. Wood. | 2 00 |
| S. R. Youngerman, | 2 00 |
| W. C. Walker, | 4 00 |
| J. W. Green, | 3 00 |
| Mitchell & Reagan, | 2 00 |
| W. H. Gifford, | 2 00 |
| R. H. Sears, | 2 00 |
| J. S. Collings, | 1 00 |
| H. E. Ellis, | 2 00 |
| **IOWA.** | |
| H. Fiske, | 1 00 |
| **OHIO.** | |
| W. H. Handford, | 2 00 |
| B. J. Smith, | 2 00 |
| **MICHIGAN.** | |
| Drs. Van Nus, | 2 00 |
| **NEW YORK.** | |
| R. B. Landon, | 1 00 |
| W. Parker, | 6 00 |
| **MINNESOTA.** | |
| W. W. Sweney, | 2 00 |

JAMES ANDREW,

### DRUGGIST AND APOTHECARY,

**West Randolph St., Chicago, Ill.**

*Dealer in Drugs, Medicines and Chemicals, Oils, Paints, Varnishes, White Lead, Dye-Stuffs and Dye-Woods.*

Medical Glass-Ware and Window Glass of all sizes. Paint, Varnish, Wall, Counter, Tooth, Hair and Clothes Brushes. Perfumery, Soaps, etc.

☞ All articles sold at this Establishment will be warranted as to quality.

CHICAGO, April, 1851.

PROSPECTUS OF THE NORTH-WESTERN

### MEDICAL AND SURGICAL JOURNAL.

---

EDITED BY

JOHN EVANS, M. D.,

PROFESSOR OF OBSTETRICS, ETC., IN RUSH MEDICAL COLLEGE, ETC., ETC.

---

This work, as heretofore, will be issued at Chicago and Indianapolis, on the first of every other month, beginning with May, in numbers each containing from 80 to 88 pages, making at the end of the year, a volume of about 500 pages, octavo, with title page and index complete.

In addition to this the NORTH-WESTERN MEDICAL INTELLIGENCER containing 16 pages, will be sent each alternate month to such subscribers as have paid all arrears and for the year advance.

As the list of contributors is annually increasing, it may be expected to furni h as good a variety of interesting original matter as any work of the kind in the country. The diseases of the West will claim especial attention.

Contributions to our pages, especially from physicians of the North-West, are earnestly solicited.

TERMS.—Two DOLLARS per annum, *in advance*, which may be paid to any of our authorized agents, or sent to us by mail, post paid, at our risk, directed to the "North-Western Medical and Surgical Journal, Chicago, Illinois." If payment be delayed until the close of the volume, three dollars will be invariably charged. No subscription received for less than an entire volume.

A few copies of the *First, Second and Third Volumes* remain unsold, and will be sent by mail for ONE DOLLAR PER. VOLUME; or the work will be furnished, half-bound, in sheep, for $1 25 per volume.

☞ Letters must be post paid to receive attention.

☞ A list of receipts will be published in each number.

☞ *Travelling Agents.*—Dr. Edmund T. Spotswood and James Gregory, for Indiana and South-Eastern Illinois; M. M. Hooten and D. L. Wood, for Illinois, Iowa, and Western Wisconsin; W. F. Lewis, for Eastern and Northern Wisconsin.

